# Simultaneous targeting of interleukin-1 and interleukin-18 is required for protection against inflammatory and septic shock

**DOI:** 10.1186/cc14023

**Published:** 2014-12-03

**Authors:** T Vanden Berghe, D Demon, P Bogaert, B Vandendriessche, A Goethals, B Depuydt, M Vuylsteke, R Roelandt, E Van Wonterghem, J Vandenbroecke, SM Choi, E Meyer, S Krautwald, W Declercq, N Takahashi, A Cauwels, P Vandenabeele

**Affiliations:** 1 Inflammation Research Center, VIB, Ghent, Belgium; 2 Department of Biomedical Molecular Biology, Ghent University, Ghent, Belgium; 3 Department of Plant Systems Biology, VIB, Ghent, Belgium; 4 Lab Aquaculture & Artemia Reference Center, Faculty Bioscience Engineering, Ghent University, Belgium; 5 Department of Nephrology and Hypertension, University Medical Center Schleswig-Holstein UKSH, Kiel, Germany

## Introduction

Sepsis is one of the leading causes of death around the world. The failure of clinical trials to treat sepsis demonstrates that the molecular mechanisms are multiple and still insufficiently understood. The objective is to clarify the long disputed hierarchical contribution of several central inflammatory mediators, namely IL-1β, IL-18, CASP7, CASP1 and CASP11, in septic shock, and to explore their therapeutic potential.

## Methods

LPS-induced and TNF-induced lethal shock, as well as cecal ligation and puncture (CLP), were performed in genetically or pharmacologically targeted mice. Body temperature and survival were monitored closely, and plasma was analyzed for several markers of cellular disintegration and inflammation.

## Results

Interestingly, deficiency of both IL-1β and IL-18 additively prevented LPS-induced mortality. The detrimental role of IL-1β and IL-18 was confirmed in mice subjected to a lethal dose of TNF, or to a lethal CLP procedure. Although their upstream activator, CASP1, and its amplifier, CASP11, are considered potential therapeutic targets because of their crucial involvement in endotoxin-induced toxicity, CASP11 or CASP1/11 deficient mice were not, or hardly, protected against a lethal TNF or CLP challenge. In line with our results obtained in genetically deficient mice, only the combined neutralization of IL-1 and IL-18, using the IL-1 receptor antagonist Anakinra and anti-IL-18 antibodies, conferred complete protection against endotoxin-induced lethality.

## Conclusion

Our data point towards the therapeutic potential of neutralizing IL-1 and IL-18 simultaneously in sepsis, rather than inhibiting the upstream inflammatory caspases.

